# Controllable ultraslow optical solitons in a degenerated two-level atomic medium under EIT assisted by a magnetic field

**DOI:** 10.1038/s41598-020-72256-4

**Published:** 2020-09-17

**Authors:** Dong Hoang Minh, Nga Luong Thi Yen, Khoa Dinh Xuan, Bang Nguyen Huy

**Affiliations:** 1 Ho Chi Minh City University of Food Industry, Ho Chi Minh City, Vietnam; 2 Vinh University, 182 Le Duan Street, Vinh City, Vietnam

**Keywords:** Slow light, Solitons

## Abstract

We proposed a simple model for generation of controllable ultraslow optical solitons of a weak probe laser light in a degenerated two-level atomic medium under electromagnetically induced transparency assisted by a magnetic field. It is shown that bright and dark optical solitons can be formed from a probe light with controllable ultraslow group velocities at a few m/s by tuning the strength of a coupling light and/or the magnetic field. In addition to the ultraslow velocity, the advantage of this model is to use a sole laser for delivering both pump and probe lights. Furthermore, one can switch between bright and dark solitons by reversing the direction of the magnetic field. Such controllable ultraslow solitons are interested in finding applications in optical communications and optical data processing.

## Introduction

Optical soliton formation is a fundamental phenomenon in nonlinear optics that attracts a great attention over the last four decades due to its potential applications^1–3^. Recently, gaseous atomic media has been the interesting subject for soliton formation since the advent of EIT^4–6^. In addition to a large suppression of optical absorption, EIT medium reduces significantly group velocity of optical lights^7,8^, supports for optical switching and bistability at low-light intensity^9,10^.

In the conventional methods for generating optical solitons, there often need intense electromagnetic fields or ultrashort laser pulses due to small nonlinearity of atomic medium for the far-off-resonance excitation frequencies. Contrastively, in the EIT atomic medium, the frequency of the interacting fields closes to atomic transition, thus the nonlinearity is enhanced significantly^11–13^. This is particularly interesting because ultraslow propagation can be achieved at a weak field intensity with controllable group velocity.

Up to date, most of works of slow light propagation and optical soliton formation in the EIT atomic medium focused on the three-level^14–26^, four-level^27–33^, and five-level^34,35^ systems in which experimental observations of several soliton types were demonstrated^24–26^. Despite of extensive proposals in this topic for multi-level atoms in which all interacting fields must be controlled synchronously, the two-level system is particularly interesting because of its simple realization. Furthermore, the previous studies often neglect degeneration of Zeeman levels which should be considered when the atoms placed in external magnetic fields or in the polarized optical fields. In this work, we propose a simple model for manipulating ultraslow-light solitons in a degenerated two-level system under EIT assisted by a static magnetic field.

In “Theoretical model” section, we describe the theoretical model based on the Maxwell-Schrödinger equations (MSE) for the evolution of atom–field interaction. “Results and analysis” section discusses the property of ultraslow bright and dark solitons with variation of the magnetic field. A possible experimental realization for the proposed model is presented in “Possible experimental realization” section. Finally, conclusions of the present work are given in “Conclusions” section.

## Theoretical model

**Figure 1:**
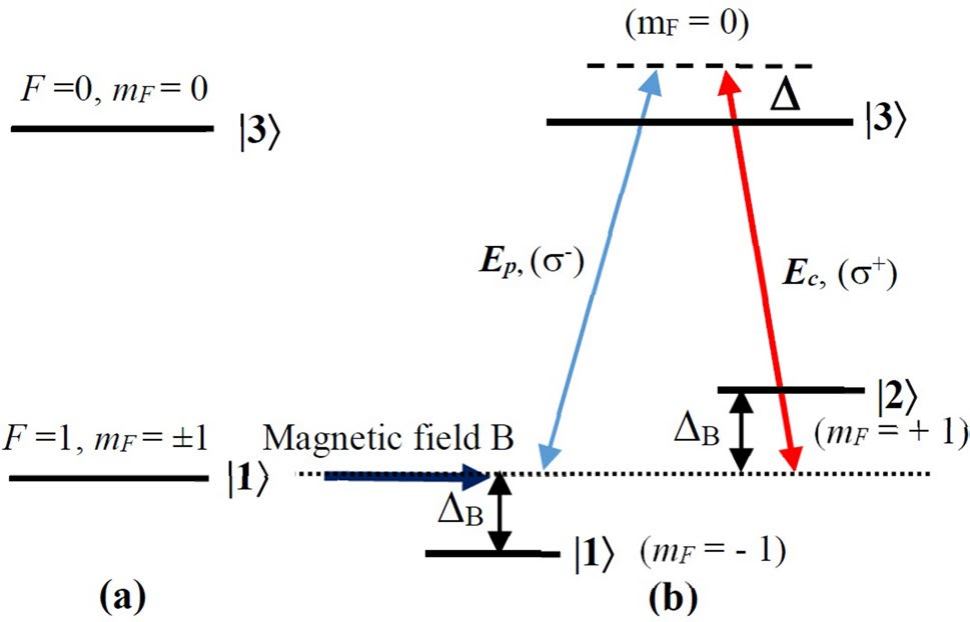
Transformation from a degenerated two-level (a) to a three-level lambda (b) configuration under a static magnetic field and two coupling and probe laser fields.

We consider a degenerated two-level atomic system consists of an upper non-degenerated level (corresponds to hyperfine state *F* = 0 with magnetic quantum number *m*_*F*_ = 0) and a lower degenerated level (correspond to hyperfine state *F* = 1 with *m*_*F*_ =  ± 1), as shown in Fig. 1a. The atomic medium is placed in a longitudinal magnetic field *B* that removes the degeneracy of the lower states with the Zeeman shifts $\pm \Delta_{B} = \mu_{B} m_{F} g_{F} B/\hbar$, where *μ*_*B*_ is the Bohr magneton, *g*_*F*_ is the Landé factor (Fig. 1b). All the atoms assumed to be optically pumped to the states |1〉 and |2〉 with the same populations, i.e. *ρ*_11_ = *ρ*_22_ = 1/2. A weak probe laser field *E*_*p*_ with the left-circularly polarized component σ^−^ (with frequency *ω*_*p*_ and one-half Rabi-frequency $\Omega_{p} = \mu_{21} E_{p} /2\hbar$) drives the transition |1〉 to |3〉. At the same time, a strong coupling laser field *E*_*c*_ with the right-circularly polarized component σ^+^ (with frequency ω_c_ and one-half Rabi-frequency $\Omega_{c} = \mu_{23} E_{c} /2\hbar$) is introduced to couple the transition between the states |2〉 and |3〉. The decay rate from the states |3〉 to |1〉 and |2〉 is given by γ. The relaxation rates of coherence between the ground states |1〉 and |2〉 by collisions are neglected.

Using the rotating-wave and the electric dipole approximations, the interaction Hamiltonian of system in the interaction picture can be written as (in the units of $\hbar$):

1 $$ H_{{\text{int}}} = 2\Delta_{B} \left| {\left. 2 \right\rangle \left. {\left\langle 2 \right.} \right|} \right. + \left( {\Delta_{B} - \Delta } \right)\left| {\left. 3 \right\rangle } \right.\left. {\left\langle 3 \right.} \right| - \left( {\Omega_{p}^{{}} \left| {\left. 3 \right\rangle } \right.\left. {\left\langle 1 \right.} \right| + \Omega_{c}^{{}} \left| {\left. 3 \right\rangle } \right.\left. {\left\langle 2 \right.} \right|} \right) + H.c $$

where $\Delta_{p} = \omega_{p} - \omega_{31} + \Delta_{B}$, $\Delta_{c} = \omega_{c} - \omega_{32} - \Delta_{B}$, and $\Delta_{p} = \Delta_{c} = \Delta$, are detuning of the probe field and coupling field from the atomic transition frequencies, respectively.

In the interaction picture, by using the time-dependent Schrödinger equations, the probability amplitudes equations for the relevant states are given by

2a $$ \frac{{\partial A_{2} }}{\partial t} = - i2\Delta_{B} A_{2} + i\Omega_{c}^{*} A_{3} , $$

2b $$ \frac{{\partial A_{3} }}{\partial t} = i\left( {\Delta - \Delta_{B} + i\gamma } \right)A_{3} + i\Omega_{p} A_{1} + i\Omega_{c} A_{2} , $$

2c $$ \left| {A_{1} } \right|^{2} + \left| {A_{2} } \right|^{2} + \left| {A_{3} } \right|^{2} = 1, $$

where *A*_*n*_ (*n* = 1, 2 3) represents amplitude of atomic wave function for each state, γ is decaying rate of the states |3〉.

Under the slowly varying envelope and rotating-wave approximations, evolution of the probe field is represented by the following wave Eq.^9^:

3 $$ \frac{{\partial \Omega_{p} }}{\partial z} + \frac{1}{c}\frac{{\partial \Omega_{p} }}{\partial t} = i\kappa_{13} A_{3} A_{1}^{*} , $$

here $\kappa_{13} = 2N\omega_{p} \left| {\mu_{31} } \right|^{2} /\left( {\hbar c} \right)$ is the propagation constant, with *N*, *μ*_13_, *c*, and *ε*_0_, are the atomic density, dipole moment between levels |1〉 and |3〉, vacuum speed of light, and vacuum dielectric constant, respectively.

## Results and analysis

In this section, we focus on interplay between the dispersion and nonlinear effects in the atomic system which can form solitons^19,29^. Firstly, we consider the dispersion properties of the atomic system by using perturbation treatment to the first order of weak probe field Ω_*p*_ while keeping all orders due to control field Ω_*c*_. To attain this aim, the perturbation approach is applied to the atomic part in terms of the expansion $A_{n} = \sum\nolimits_{k} {A_{n}^{\left( k \right)} }$, where $A_{{}}^{\left( k \right)}$ is the *k*-th order part of $A_{n}^{{}}$ in the probe field Ω_*p*_. To the first-order of the probe field Ω_*p*_, we assume that the atomic is initially in the ground states |1〉 and |2〉 with $A_{1}^{(0)} \simeq A_{2}^{(0)} \simeq 1{/}2$ and $A_{3}^{\left( 0 \right)} = 0$. By performing the time Fourier transform of Eqs. (2) and (3) and keeping up to the first order of Ω_*p*_, we obtained

4a $$ \left( {\omega - 2\Delta_{B} } \right)a_{2}^{\left( 1 \right)} + \Omega_{c}^{*} a_{3}^{\left( 1 \right)} = 0, $$

4b $$ \left( {\omega + \Delta - \Delta_{B} + i\gamma } \right)a_{3}^{\left( 1 \right)} + \Omega_{c} a_{2}^{\left( 1 \right)} = - \frac{1}{2}\Lambda_{p} , $$

4c $$ \frac{{\partial \Lambda_{p} }}{\partial z} - \frac{i\omega }{c}\Lambda_{p} = i\kappa_{13} a_{3}^{\left( 1 \right)} , $$

here $a_{n}^{\left( 1 \right)}$(n = 1, 2, 3) and Λ_p_ are the Fourier transforms of $A_{n}^{\left( 1 \right)}$ and Ω_p_ respectively, and *ω* is the Fourier variable.

**Figure 2:**
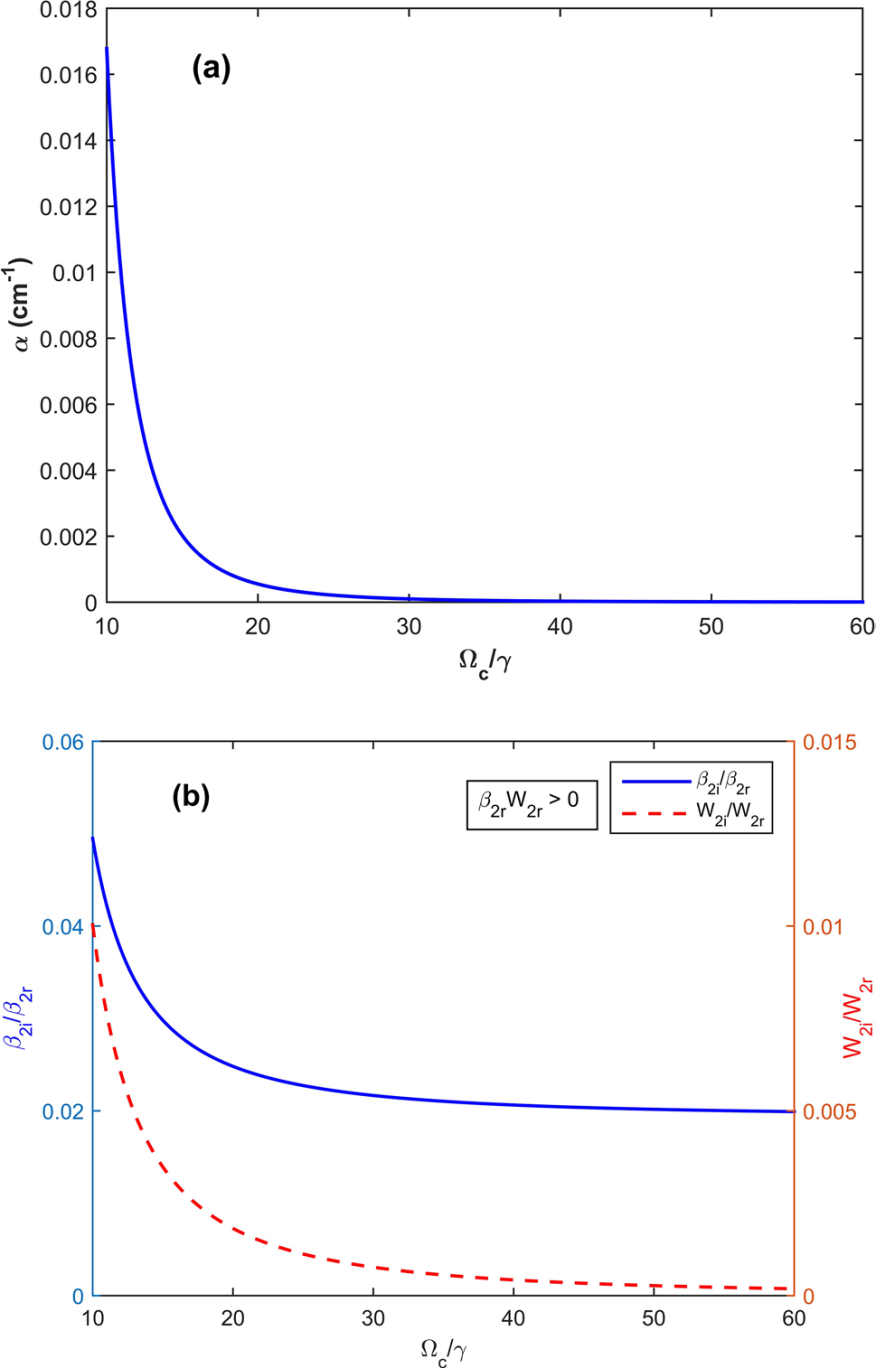
(**a**) The absorption coefficient α; (**b**) the ratios of the imaginary and real parts of the coefficients *β*_2*i*_/*β*_2*r*_ (*solid*) and *W*_*i*_/*W*_*r*_ (*dashed*) versus the dimensionless Rabi frequency Ω_c_/γ. This case corresponds to condition of bright solitons (*β*_2*r*_.*W*_*r*_ > 0).

**Figure 3:**
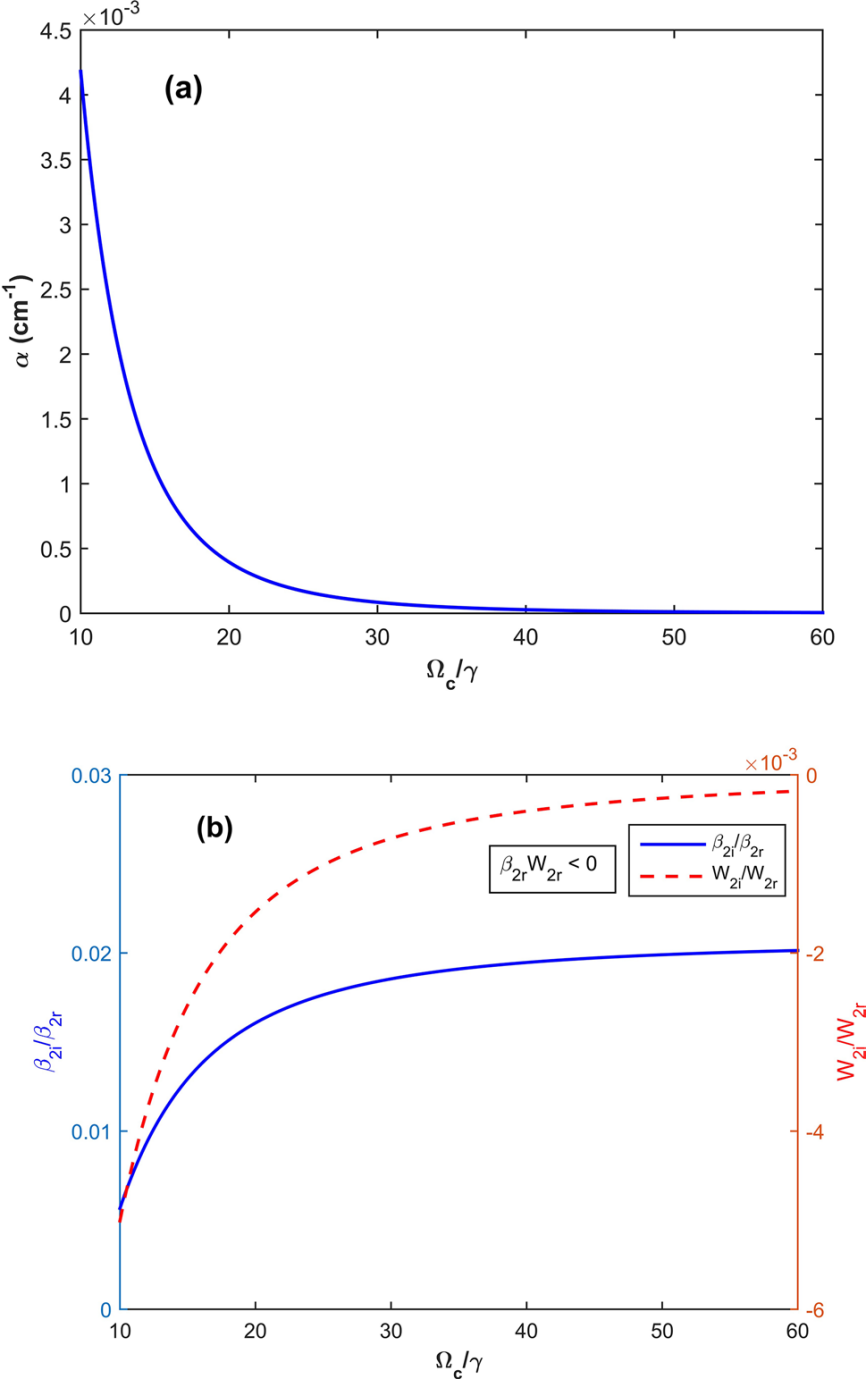
(**a**) The absorption coefficient α; (**b**) the ratios *β*_2*i*_/*β*_2*r*_ (*solid*) and *W*_*i*_/*W*_*r*_ (*dashed*) versus Ω_c_/γ with the parameters as same as those in Fig. 2 except for Δ_*B*_ = 0.33γ or *B* = 0.33*γ*_*c*_. This case corresponds to the dark solitons (*β*_2*r*_.*W*_*r*_ < 0).

By solving Eq. (4c) with a substitution form Eqs. (4a) and (4b), we obtain solution for the probe field

5 $$ \Lambda_{p} \left( {z,\omega } \right) = \Lambda_{p} \left( {0,\omega } \right)\exp \left[ {i\beta \left( \omega \right)z} \right], $$

where *β*(*ω*) is the propagation constants denoted by

6 $$ \beta \left( \omega \right) = \frac{\omega }{c} + \frac{{\left( {\omega - 2\Delta_{B} } \right)\kappa_{13} }}{{2\left| {\Omega_{c} } \right|^{2} - 2\left( {\omega - 2\Delta_{B} } \right)\left( {\omega + \Delta - \Delta_{B} + i\gamma } \right)}} = \beta_{0} \left( 0 \right) + \beta_{1} \left( 0 \right)\omega + \frac{1}{2}\beta_{2} \left( 0 \right)\omega^{2} + O\left( {\omega^{3} } \right), $$

with

7a $$ \beta_{0} \left( 0 \right) = - \frac{{2\kappa_{13} \Delta_{B} }}{2D}, $$

7b $$ \beta_{1} \left( 0 \right) = \left. {\frac{d\beta \left( \omega \right)}{{d\omega }}} \right|_{\omega = 0} = \frac{1}{c} + \frac{{\kappa_{13} \left( {\Omega_{c}^{2} + 4\Delta_{B}^{2} } \right)}}{{2\left| D \right|^{2} }}, $$

7c $$ \beta_{2} \left( 0 \right) = \left. {\frac{{d^{2} \beta \left( \omega \right)}}{{d\omega^{2} }}} \right|_{\omega = 0} = \frac{{\kappa_{13} \left[ {\left( {\Delta - 5\Delta_{B} + i\gamma } \right)\left| {\Omega_{c} } \right|^{2} - 8\Delta_{B}^{3} } \right]}}{{2\left| D \right|^{2} D}}, $$

7d $$ D = \left| {\Omega_{c} } \right|^{2} + 2\Delta_{B} \left( {\Delta - \Delta_{B} + i\gamma } \right), $$

where *β*_0_(0), *β*_1_(0), and *β*_2_(0) relates to physics quantities. Indeed,$\beta_{0} \left( 0 \right) = \phi + i\alpha /2$ describes the phase shift *ϕ* per unit length with absorption coefficient *α* (see Figs. 2a, 3a) of the probe field; $V_{g} = {\text{Re}} \left[ {1/\beta_{1} (0)} \right]$ denotes the propagation group velocity of optical solitons; and $\beta_{2} \left( 0 \right)$ represents the group-velocity dispersion (GVD) that leads to change in probe pulse’s shape and loss of probe field intensity.

To study formation of optical solitons, there should balance the interplay between group velocity dispersion and nonlinear effects. We consider the nonlinear polarization on the right-hand sides of Eq. (4c) and take a trial function $\Omega_{p} \left( {z,t} \right) = \Omega_{p} \left( {z,t} \right)\exp \left[ {i\beta_{0} \left( 0 \right)z} \right]$ for Eq. (3), we obtain the nonlinear wave equations for the slowly varying envelope $\Omega_{p} \left( {z,t} \right)$.

8 $$ - i\left[ {\frac{\partial }{\partial z} + \beta_{1} \left( 0 \right)\frac{\partial }{\partial t}} \right]\Omega_{p} + \frac{1}{2}\beta_{2} \left( 0 \right)\frac{{\partial^{2} \Omega_{p} }}{{\partial t^{2} }} = {\text{NLT}}, $$

where NLT is a nonlinear term given by ${\text{NLT}} = - \kappa_{13} A_{3}^{\left( 1 \right)} \exp \left[ { - i\beta_{0} \left( 0 \right)z} \right]\left( {\left| {A_{2}^{\left( 1 \right)} } \right|^{2} + \left| {A_{3}^{\left( 1 \right)} } \right|^{2} } \right)$ with parameters:

9a $$ A_{2}^{\left( 1 \right)} = \frac{{ - 2\Delta_{B} }}{2D}\Omega_{p} , $$

9b $$ A_{3}^{\left( 1 \right)} = \frac{{ - \Omega_{c}^{*} }}{2D}\Omega_{p} . $$

It is convenient to transform Eq. (8) into a moving frame by changing $\xi = z$ and $\tau = t - z/V_{g}$, we obtain the following equation for Ω_*p*_:

10 $$ i\frac{\partial }{\partial \xi }\Omega_{p} - \frac{1}{2}\beta_{2} \left( 0 \right)\frac{{\partial^{2} \Omega_{p} }}{{\partial \tau^{2} }} = W\exp \left( { - \alpha \xi } \right)\left| {\Omega_{p} } \right|^{2} \Omega_{p} , $$

where absorption coefficient $\alpha = 2{\text{Im}} \left[ {\beta_{0} \left( 0 \right)} \right]$ and

11 $$ W = \frac{{\kappa_{13} \Delta_{B} \left( {\left| {\Omega_{c} } \right|^{2} + 4\Delta_{B}^{2} } \right)}}{{4\left| D \right|^{2} D}}. $$

Since the coefficients in NLS Eq. (10) are therefore complex, thus Eq. (10) generally does not have soliton solutions. However, in the presence of the coupling field, the absorption for the probe field can be suppressed under EIT conditions, where the probe field intensity relates to $\exp \left( { - \alpha l} \right) \simeq 1$ (*l* is length of the atomic medium). Furthermore, as we can see below, for the practical parameters one may find conditions so that the imaginary part of the complex coefficient in Eq. (11) much smaller than their corresponding real part, i.e., $\beta_{2} \left( 0 \right) = \beta_{2r} \left( 0 \right) + i\beta_{2i} \left( 0 \right) \simeq \beta_{2r} \left( 0 \right)$, and $W = W_{r} + iW_{i} \simeq W_{r}$. Under the regime of these parameters, we can neglect the imaginary parts and make the Eq. (11) to be integrable, then Eq. (10) can be reduced to the standard nonlinear Schrödinger equation:

12 $$ i\frac{\partial }{\partial \xi }\Omega_{p} - \frac{1}{2}\beta_{2r} \left( 0 \right)\frac{{\partial^{2} \Omega_{p} }}{{\partial \tau^{2} }} = W_{r} \left| {\Omega_{p} } \right|^{2} \Omega_{p} , $$

which admits the solutions describing various types of solitons^2,19,28,29^, such as the right ($\beta_{2r} W_{r} > 0$) and dark ($\beta_{2r} W_{r} < 0$) solitons, depending on choosing the parameters. The fundamental bright soliton is given by:

13 $$ \Omega_{p} = \Omega_{p0} sech\left( {\tau /\tau_{0} } \right)\exp \left( { - i\xi W_{r} \left| {\Omega_{p0} } \right|^{2} /2} \right), $$

where sech(*τ*/*τ*_0_) is the hyperbolic secant function, amplitude Ω_p0_ and width τ_0_ subject only to the constraint $\left| {\Omega_{p0} \tau_{0} } \right|^{2} = 2\beta_{2r} \left( 0 \right)/W_{r}$. Note that the condition $\left| {\Omega_{p0} \tau_{0} } \right|^{2} \ll \left| {\Omega_{c} \tau_{0} } \right|^{2}$ used to derive Eqs. (10) and (12) is fulfilled for weak probe field. Therefore width *τ*_0_ should be chosen to meet $2\beta_{2r} \left( 0 \right)/W_{r} \ll \left| {\Omega_{c} \tau_{0} } \right|^{2}$.

The fundamental dark soliton of Eq. (12) with $\beta_{2r} W_{r} < 0$ is given by

14 $$ \Omega_{p} = \Omega_{p0} \tanh \left( {\tau /\tau_{0} } \right)\exp \left( { - i\xi W_{r} \left| {\Omega_{p0} } \right|^{2} } \right), $$

where the envelope of the probe pulse is chosed as $\Omega_{p} \left( {\xi = 0,\tau } \right) = \Omega_{p0} tanh\left( {\tau /\tau_{0} } \right)$^37^.

We now consider practical parameters to show the existence of bright and dark solitons in the degenerated two-level atomic system. For this purpose, we plot the cases of bright and dark solitons in Figs. 2 and 3, respectively.

In Fig. 2, the absorption coefficient *α* and the ratios *β*_2*i*_/*β*_2*r*_ and *W*_*i*_/*W*_*r*_ are plotted versus the dimensionless Rabi frequency Ω_*c*_/*γ* with parameters *κ*_13_ = 1 × 10^9^ cm^−1^ s^−1^, Δ = 3 × 10^8^ s^−1^, and *γ* = 6 × 10^6^ s^−1^^,^^19,28^, Δ_*B*_ =  − 2 × 10^6^ s^−1^ =  − 0.33*γ*, which corresponds to *B* =  − 0.33*γ*_*c*_ (we note that when the Zeeman shift Δ_B_ is scaled by γ, then the magnetic strength *B* should be in unit of the combined constant $\gamma_{c} = \hbar \mu_{B}^{ - 1} g_{F}^{ - 1} \gamma$). The figure clearly demonstrates that there exists a region of the parameters in which absorption for the probe field can be almost suppressed under presence of the magnetic field^9^. In this region we see that *β*_2r_.*W*_*r*_ > 0, thus, the bright soliton can be formed.

Using the same parameters as used in Fig. 2 except for Δ_*B*_ = 2 × 10^6^ s^−1^, (corresponds to *B* = 0.33*γ*_*c*_), we plot the absorption coefficient α and the ratios *β*_2*i*_/*β*_2*r*_ and *W*_*i*_/*W*_*r*_ versus the dimensionless Rabi frequency Ω_c_/γ, as shown in Fig. 3. In this parameter regions, *β*_2*r*_.*W*_*r*_ < 0, thus, dark solitons can be admitted. From both Figs. 2 and 3 one can see a possible switching between a bright and dark soliton by reversing direction of the external magnetic field.

**Figure 4:**
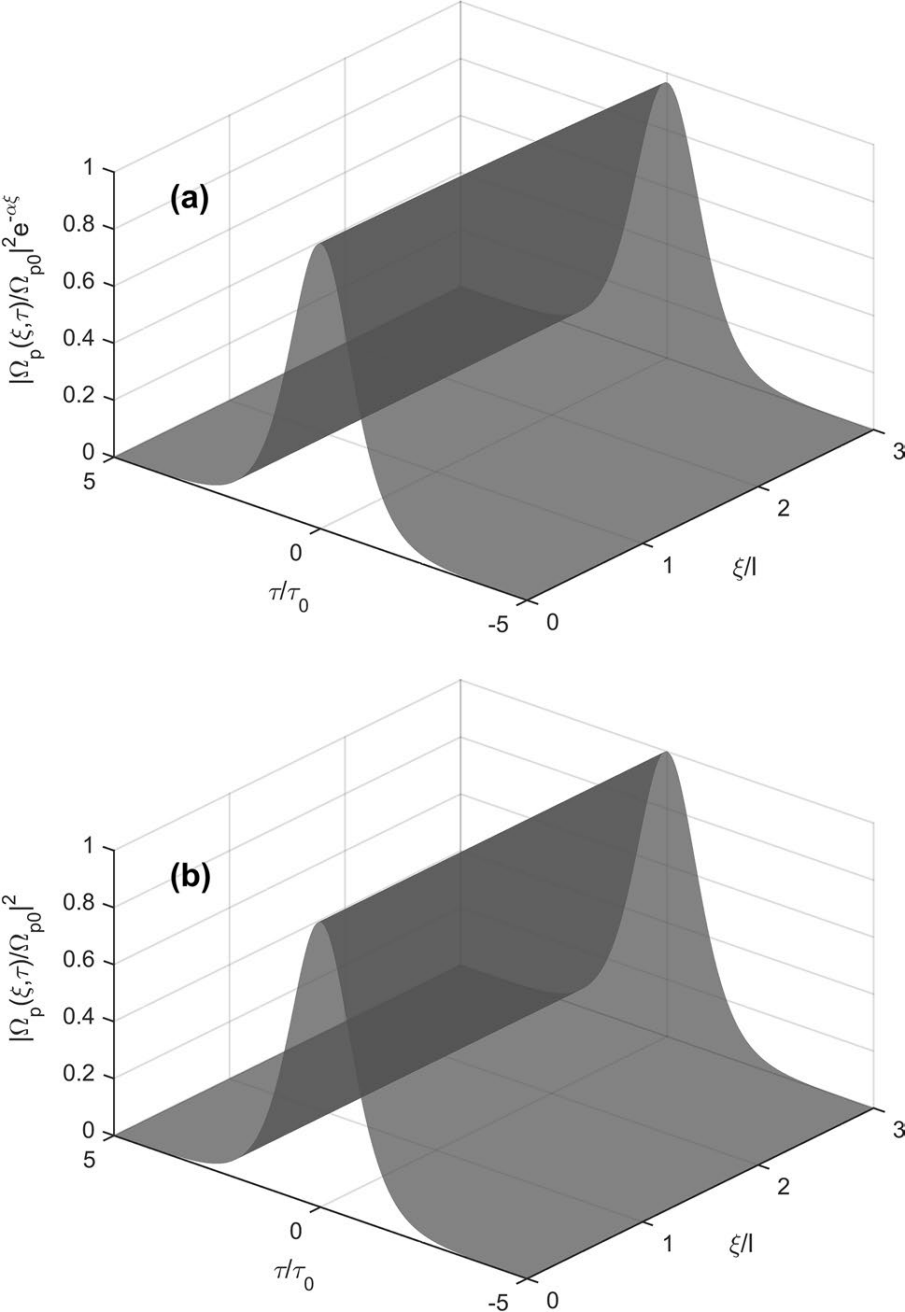
Surface plots of $\left| {\Omega_{p} /\Omega_{p0} } \right|^{2} e^{ - \alpha \xi }$ (**a**) and bright soliton $\left| {\Omega_{p} /\Omega_{p0} } \right|^{2} = sech^{2} \left( {\tau /\tau_{0} } \right)$ (**b**) versus *τ*/*τ*_0_ and propagation distance *ξ*/*l* under the boundary condition $\Omega_{p} \left( {\xi = 0,\tau } \right) = \Omega_{p0} sech\left( {\tau /\tau_{0} } \right)$; here *l* = 1 cm, *τ*_0_ = 1.0 × 10^−6^ s.

In order to further confirm formation of the bright and dark solitons as predicted in Figs. 2 and 3, we make numerical simulations directly from the Eq. (10) at the boundary condition $\Omega_{p} \left( {\xi = 0,\tau } \right) = \Omega_{p0} sech\left( {\tau /\tau_{0} } \right)$ with *τ* = 1.0 × 10^−6^ s. The evolution of $\left| {\Omega_{p} /\Omega_{p0} } \right|^{2} e^{ - \alpha \xi }$ as a function of time *τ*/*τ*_0_ and propagation distance *ξ*/*l* is shown in Fig. 4a whereas the result obtained from the standard integrable Eq. (12) with the fundamental bright soliton solution given in Eq. (13) $\left| {\Omega_{p} /\Omega_{p0} } \right|^{2} = sech^{2} \left( {\tau /\tau_{0} } \right)$ is shown in Fig. 4b. In details, with Ω_c_ = 6 × 10^7^ s^−1^, other parameters are the same as those in Fig. 2, we obtained: *ϕ* = 0.83 rad cm^−1^, *α* = 1.67 × 10^−2^ cm^−1^, *β*_2_(0) = (4.07 + 0.201*i*) × 10^−14^ s^2^ cm^−1^, *W* = (1.32 + 0.0132*i*) × 10^−16^ s^2^ cm^−1^. This case shows $\beta_{2r} .W_{r} > 0$, namely, bright solitons can be formed. Notice that the imaginary parts of these parameters are much smaller than their corresponding real parts. Furthermore, the bright soliton propagates with the group velocity *V*_g_/*c* = 1.05 × 10^−4^ that is much smaller than the vacuum light speed *c*, i.e., subluminal propagation. As shown in Fig. 4a, the probe field propagates without loss, therefore, subluminal bright solitons can be maintained in the medium.

**Figure 5:**
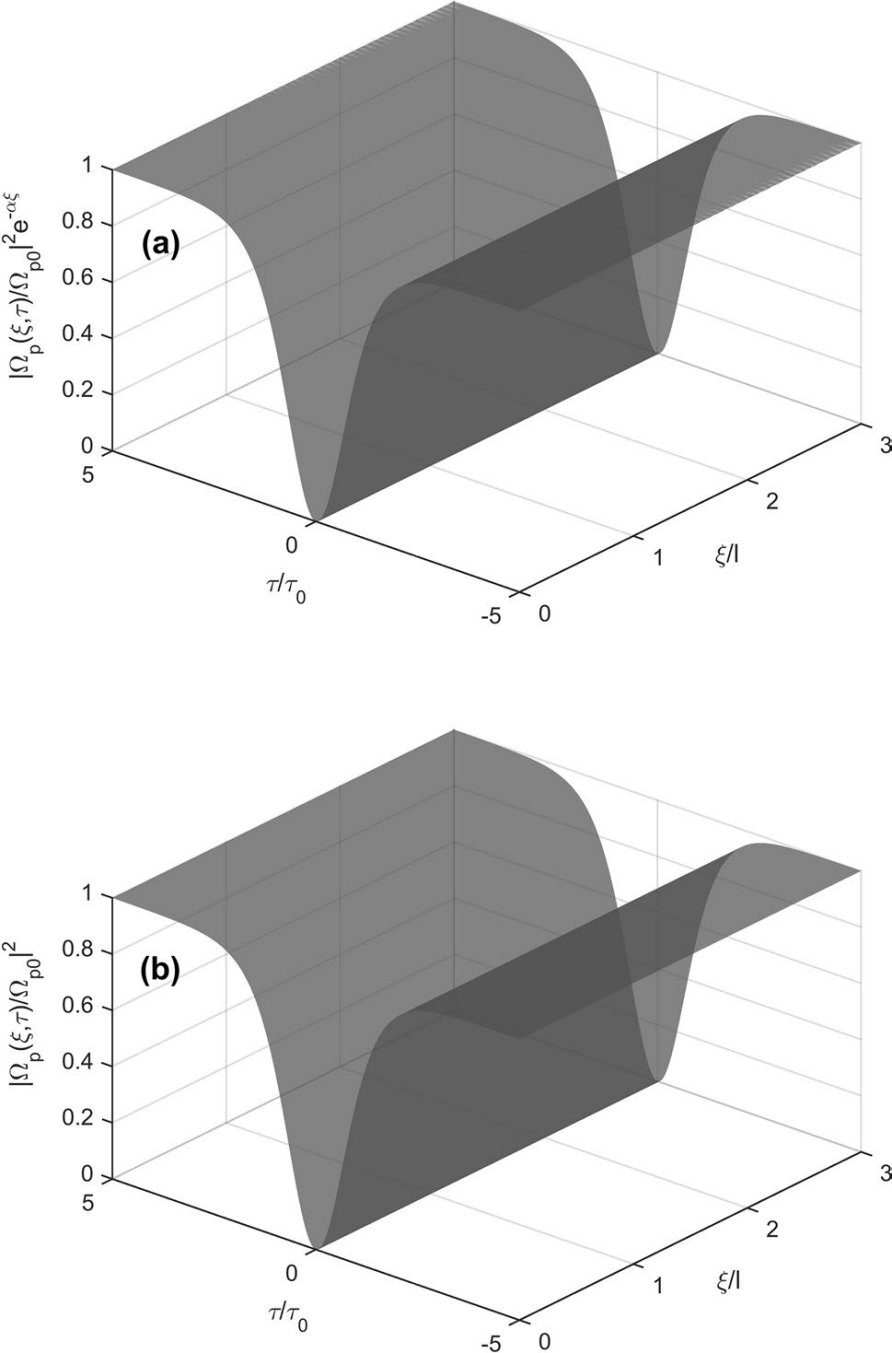
Surface plots of $\left| {\Omega_{p} /\Omega_{p0} } \right|^{2} e^{ - \alpha \xi }$ (**a**) and dark soliton $\left| {\Omega_{p} /\Omega_{p0} } \right|^{2} = \tanh^{2} \left( {\tau /\tau_{0} } \right)$ (**b**) versus *τ*/*τ*_0_ and propagation distance *ξ*/*l* with the boundary condition $\Omega_{p} (\xi = 0,\tau ) = \Omega_{p0} tanh(\tau {/}\tau_{0} )$, and *l* = 1 cm, *τ*_0_ = 1.0 × 10^−6^ s.

Now we consider influence of reversing direction of the magnetic field, as same as above case, namely, Δ_B_ = 2 × 10^6^ s^−1^. In this case we obtain *V*_*g*_/*c* = 4.23 × 10^−4^, *ϕ* =  − 0.41 rad cm^−1^, *α* = 4.18 × 10^−3^ cm^−1^, *β*_2_(0) = (4.74 + 0.026i) × 10^−15^ s^2^ cm^−1^, *W* = (− 1.64 + 0.0082i) × 10^−17^ s^2^ cm^−1^. These results lead to $\beta_{2r} .W_{r} < 0$, namely, the dark soliton can be formed as shown in Fig. 5. It is worth to note that the given parameter values lead to a negligible loss for both the dark solitons (Fig. 5). Furthermore, the reversion of magnetic direction leads to switching between dark and bright solitons.

**Figure 6:**
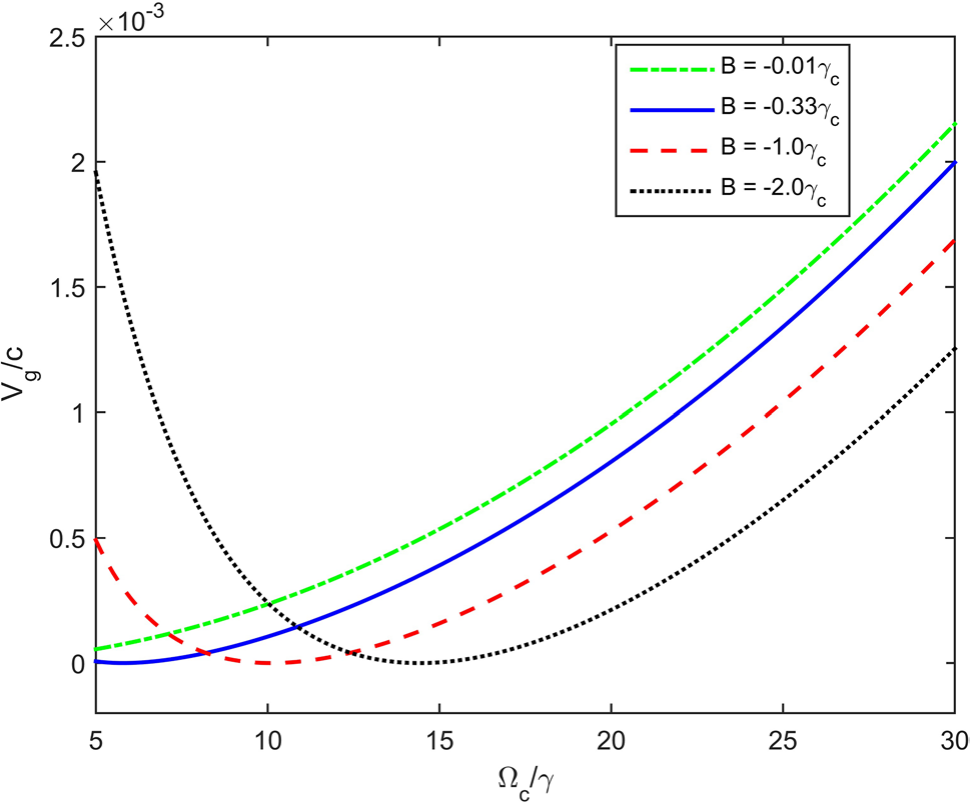
Dependence of velocity of the probe light versus the Rabi frequency Ω_c_ at different values of the magnetic field.

**Figure 7:**
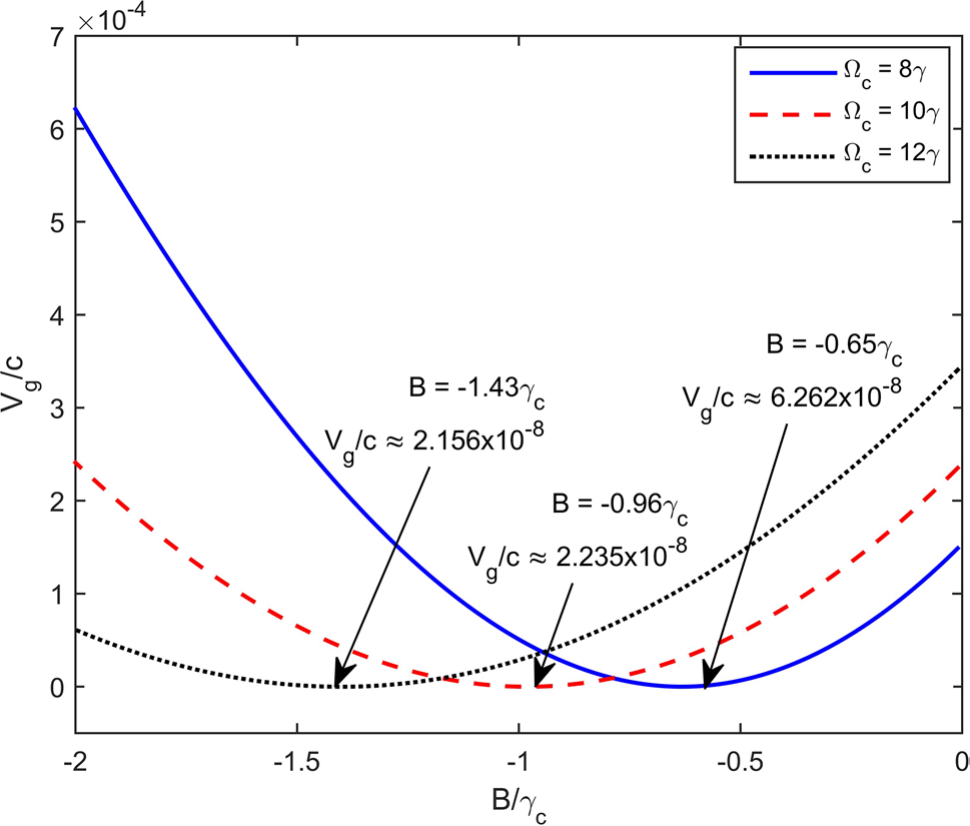
Variation of group velocity versus magnetic strength at different values of the coupling light.

Finally, we consider influences of the coupling light and magnetic strength on the group velocity of the probe light by plotting *v*_*g*_/*c* versus Rabi frequency Ω_*c*_ and versus magnetic strength, as shown in Figs. 6 and 7, respectively. Here other parameters are chosen as *κ*_13_ = 1 × 10^9^ cm^−1^ s^−1^, Δ = 3 × 10^8^ s^−1^, γ = 6 × 10^6^ s^−1^^36^. The results in Figs. 6 and 7 show that, for a given coupling strength (or magnetic strength), it could be possible to choose an optimum magnetic strength (or coupling strength) to get smallest group velocity. Indeed, one may slow probe pulse down to 6.5 m/s which is the same order with experimental realization^7^.

## Possible experimental realization

In this section, we discuss a possible experimental realization for the case of ^87^Rb atoms on the 5S_1/2_ ↔ 5P_3/2_ transitions. Here, the states |1〉, |2〉, and |3〉 are given as 5S_1/2_ (F = 1, m_F_ =  − 1), 5S_1/2_(F = 1, m_F_ = 1), and 5P_3/2_ (F = 0, m_F_ = 0), respectively (see Fig. 1). Both the probe and coupling fields can be delivered by a sole laser working at 780 nm. The atomic medium can be produced in a vapor cell placed inside a solenoid tube connected with a variation DC current source via interchangeable anode–cathode switch. This configuration can deliver controllable magnetic field in both magnitude and direction.

In order to ensure selection rules for the excitation configuration, the coupling and probe beams are directed to quarter-wave plates to produce circularly polarized beams (see Fig. 1) where both of which propagate in opposite directions. The generation of dark solitons can be used the trapezoidal optical integrator whereas detection of the probe pulse can be used by the first-derivative optical differentiator and the first-order Butterworth detector^37^.

## Conclusions

We have proposed a simple model for generation of tunable ultraslow optical solitons of a weak probe laser pulse in a degenerated two-level atomic medium under an external magnetic field. The system can generate and switch between bright and dark solitons by reversing the direction of the external magnetic field. Furthermore, the solitons can be controlled to propagate with ultraslow group velocity by tuning the strength of the coupling light and/or the magnetic field. In addition to the ultraslow velocity, the advantage of this model is to use a sole laser for delivering both pump and probe lights. Such tunable optical solitons are interesting for finding applications in optical information processing and logic gates.

## Methods

Using a method of multiple scales with amplitude variable approach we derive the nonlinear Schrödinger equation that governs the time evolution of probe pulse envelope. The formation, evolution and dynamics of the ultraslow optical soliton by using a standard soliton perturbation theory. All analytical predicts are checked by numerical simulations in the MATLAB.
